# Involvement of CircRNA Expression Profile in Diabetic Retinopathy and Its Potential Diagnostic Value

**DOI:** 10.3389/fgene.2022.833573

**Published:** 2022-02-17

**Authors:** Hengqian He, Juntao Zhang, Weikun Gong, Mengyun Liu, Hao Liu, Xiaoyong Li, Yufei Wu, Qinkang Lu

**Affiliations:** ^1^ Department of Ophthalmology, The Affiliated People’s Hospital of Ningbo University, Ningbo, China; ^2^ Center for Disease Control and Prevention of Yinzhou District, Ningbo, China

**Keywords:** circular RNAs, diabetic retinopathy, diabetes mellitus, biomarker, diagnostic value

## Abstract

**Background:** Circular RNAs (circRNAs), a class of non-coding and undegradable RNAs, play many pathological functions by acting as miRNA sponges, interacting with RNA-binding proteins, and others. The recent literature indicates that circRNAs possess the advanced superiority for the early screening of diabetic retinopathy (DR).

**Methods:** CircRNA sources of peripheral blood mononuclear cells (PBMCs) from healthy controls (*n* = 4), diabetes mellitus patients (DM) (*n* = 4), and DR patients (*n* = 4) were extracted for circular RNA microarray analysis. Enriched biological modules and signaling pathways were analyzed by Gene Ontology Enrichment and Kyoto Encyclopedia of Genes and Genomes analysis, respectively. Real-time quantitative reverse transcription PCR (RT-qPCR) was performed to validate differentiated levels of several circRNAs (fold change ≥2, *p* < .05) in different groups of healthy control subjects (*n* = 20), DM patients (*n* = 60), and DR patients (*n* = 42). Based on our clinical data from DR, the diagnostic performance of candidate circRNAs was measured by operating characteristic curves (ROCs). Subsequently, their circRNA–miRNA networks were constructed by bioinformatics analysis.

**Results**: Circular RNA microarray analysis was performed, and 2,452 and 289 circRNAs were screened with differential expression in DR patients compared to healthy controls and DM patients, respectively. Enrichment analyses showed that circRNAs in DR patients were enriched in extracellular matrix (ECM)–receptor interaction and focal adhesion pathways. The top 5 differential circRNAs in circRNA microarray analysis were subsequently quantified and verified by RT-qPCR. Consistently, a significant 2.2-fold reduction of hsa_circ_0095008 and 1.7-fold increase in hsa_circ_0001883 were identified in DR patients compared to DM patients. Meanwhile, the area under curves of hsa_circ_0095008 and hsa_circ_0001883 were 0.6710 (95% CI, 0.5646–0.7775) (*p* = 0.003399) and 0.6071 (95% CI, 0.4953–0.7189) (*p* = 0.06644), respectively, indicating a good diagnostic value.

**Conclusion:** Our study provided a new sight for the pathological mechanism of DR and revealed the potential value of hsa_circ_0095008 and hsa_circ_0001883 as diagnostic biomarkers for the early diagnosis of DR patients.

## Introduction

The incidence of diabetes mellitus (DM) has increased dramatically worldwide in recent years, ranking as the ninth leading cause of death. Of these, 90% of the patients had type 2 diabetes mellitus (T2DM) (Zheng et al., 2018). Unhealthy dietary habits, lifestyle, and genetic factors were involved in the development of T2DM (Kautzky-Willer et al., 2016). It is reported that the vast majority of DM have at least one complication, including diabetic nephropathy, cardiovascular disease, and diabetic retinopathy (DR) (Naqshbandi et al., 2008; Gu et al., 2020). As a common complication of DM, DR is the main cause of impaired vision in diabetic patients. Retinal microvascular leakage and obstruction were attributed to fundus lesions, macular edema, and others (Gonzalez-Casanova et al., 2021). Currently, vascular endothelial growth factor A (VEGFA) inhibitors are the only drugs in clinical therapy that can effectively treat DR (Antonetti et al., 2021). Furthermore, vascular endothelial growth factor A (VEGFA) inhibitors are not effective in all patients with DR (Funatsu et al., 2009).

Circular RNAs (circRNAs) have become a new hot spot in the field of non-coding RNA research apart from microRNA (miRNA) and long non-coding RNA (Cai et al., 2019). CircRNAs are widely distributed in eukaryotic cells and participate in the pathogenesis and development of multiple types of diseases, including cancers, neurological diseases, and others (Altesha et al., 2019; Mengxue Xu et al., 2020; Lin et al., 2020; Wu et al., 2020; Fang et al., 2021). CircRNAs regulate various cellular activities via affecting RNA polymerase prolongation, acting as miRNA sponges to regulate target gene expression, and interacting with RNA-binding proteins to regulate the translation process (Zhang et al., 2013; Ebbesen et al., 2017; Hsiao et al., 2017). Increasing evidence shows that circRNAs are closely related to a variety of human diseases, such as tumors and DR (Wang et al., 2017; Zhang et al., 2017). For example, circHIPK3, circRNA cZNF609, and hsa_circ_0005015 have been proven to play a vital role in the progression of DR by regulating the growth, proliferation, migration, and tube formation of retinal vascular endothelial cells (Liu et al., 2017; Shan et al., 2017). Furthermore, previous studies identified that circRNA such as circular RNA-ZNF532 and circ-PSEN1 regulate DR progression by serving as miRNA sponges (Jiang et al., 2020; Zhu et al., 2021).

CircRNAs are universally expressed and conserved in human and vertebrate neural retina (Sun et al., 2019; Meng-Lan Li et al., 2021). In mouse and rat retinas, circRNA population increases during development, with significant developmental stage specificity (Han et al., 2017; George et al., 2019; Mellough et al., 2019; Kaining Chen et al., 2021; Gang Chen et al., 2021). CircRNAs are aberrantly expressed in retina-related diseases (Wang et al., 2018; Cao et al., 2019; Chen et al., 2020a; Sun et al., 2020; Sun et al., 2021). Furthermore, expression abnormalities of circRNAs appear earlier than the disease onset in a retinal degeneration model (Chen et al., 2020b).

Based on the prevailing biological functions of circRNAs, it suggests that circRNAs may be an ideal molecular marker for DR diagnosis and therapeutic targets. To investigate the circRNAs associated with DR occurrence, we analyzed circRNA expression profiles in healthy controls, DM, and DR patients, followed by the verification of differential expressions of circRNAs by RT-qPCR.

## Materials and Methods

### Peripheral Blood Mononuclear Cell Collection

Peripheral blood mononuclear cells (PBMCs) were obtained from 60 cases of DM patients, 42 cases of DR patients and 20 cases of healthy individuals by using anticoagulation tubes. Ficoll-Paque PLUS (GE Healthcare, United States) was added in blood samples with an equal amount of PBS. The mixture was centrifuged at 1,500 rpm for 40 min, and then in the middle, PBMCs were washed with PBS. Finally, PBMCs were kept at –80°C in TRIzol (Sigma-Aldrich, United States) for subsequent use. All participants agreed and signed the informed consent. The study was approved by the Research Ethics Board (REB) of the Affiliated People’s Hospital of Ningbo University (approval number 2019–048).

### Total RNA Extraction and Quantitative Real-Time PCR

Total RNA was extracted from PBMCs using TRIzol (Sigma-Aldrich, United States) and then reverse transcribed into cDNA RNA using PrimeScript^TM^ RT reagent (Takara, United States). CircRNAs were quantified by qRT-PCR using Power SYBR Green Master Mix (Applied Biosystems, United States) on 7500 Fast Real-Time PCR System (Applied Biosystems, United States), according to manufacturer’s instructions. Primer sequences of all circRNAs are listed in Table1.

**TABLE 1:** Primers of validated circRNAs in RT-qPCR.

circRNA ID	Forward (5–3′)	Reverse (5–3′)
hsa_circ_0095008	ATG​CGA​CCA​TCC​ACC​TCA​AAG	ACA​TCA​CAC​ACA​ATC​ACG​GCA
hsa_circ_0005062	TCA​TCA​GCA​CCC​TGT​CGT​CT	CTG​CTT​TTC​CTG​TGA​TTT​TAC​CCA
hsa_circ_0001883	AGA​GAG​TAC​CAG​ACC​CGA​CA	GCA​AGT​GAG​CGA​AAT​GCT​CTT
hsa_circ_0040707	GCT​CTT​TGC​AGG​GTC​GAC​AA	AGT​GGT​TTT​TGG​GGC​CGT​TG
hsa_circ_0002031	GTG​ATC​GTT​GGC​GGA​CAT​TT	ATG​CTG​CTG​TCA​TGT​GCT​TCT
GAPDH	ATG​GAA​ATC​CCA​TCA​CCA​TCT​T	CGCCCCACTTGATTTTGG

### Circular RNA Microarray Analysis

For circRNA microarray analysis, the total RNA sample was isolated using TRIzol (Sigma-Aldrich, United States) and then purified by an miRNA Isolation Kit (MACHEREY- NAGEL, Cat#740955, Germany), according to the manufacturer’s instructions. The extracted RNA was subsequently amplified and labeled with an Ambion WT Expression Kit (Cat#740955, Ambion, United States). Labeled samples were dissolved in a hybridization solution to load onto a Capital Bio Technology Human CircRNA Array v2 microarray (Agilent, United States) overnight. The circRNA microarray results were analyzed by Agilent GeneSpring software. The circRNAs with fold change ≥2 and *p* ≤ 0.05 were considered as upregulated or downregulated in circRNA microarray analysis.

### CircRNA–miRNA Network Prediction

miRanda-3.3 software was used to predict circRNA-targeting miRNAs based on the degree of sequence complementarity between miRNAs and circRNAs. These circRNA–miRNA pairs were combined at entropy values below 20 and then constructed into networks using the open source bioinformatics software Cytoscape (v3.19.0, Institute of Systems Biology, United States).

### Statistical Analysis

SPSS version 13.0 (SPSS Inc, IL, United States) was utilized for statistical analysis of all data in this study. The significant difference of RT-qPCR results was analyzed by using Student’s *t* test. For the comparison of each group, *p* < 0.05 was considered statistical significant.

## Results

### CircRNA Expression Profiles in PBMCs of Different Groups

**FIGURE 1:**
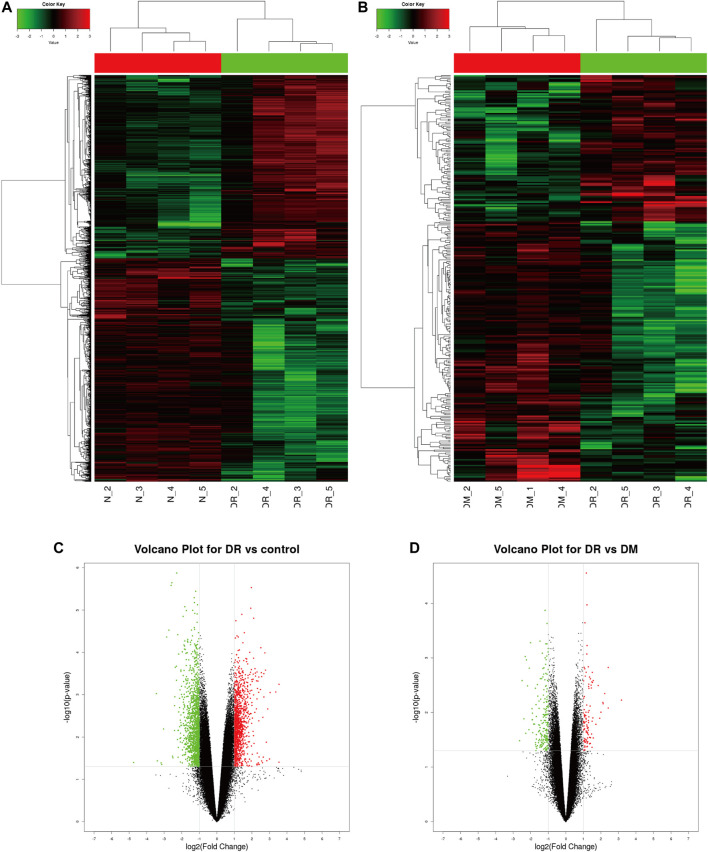
Profile of circRNA level in DR samples. **(A)** Identified circRNAs between DR and control groups by heat map analyzes. **(B)** Identified circRNAs between DR and DM groups by heat map analyzes. **(C)** Differentially expressed circRNAs between DR and control groups by Volcano map analysis; **(D)** The differently expressed circRNAs between DR and DM groups by Volcano map analysis. The red dots represent significant upregulation, and green dots represent significant downregulation.

PBMC samples were obtained and extracted from healthy controls and DM patients (with or without DR). Human CircRNA microarray v2 (Capital Bio Technology) was performed to detect the profile of circRNA expression, which revealed significant differences in DR patients compared to healthy controls (Figure 1A) or DM patients (Figure 1B) by hierarchical clustering. Volcano plot filtering was used to represent significant changes in differential circRNAs (FC ≥ 2 and *p* ≤ 0.05) between two groups. The results confirmed that a total of 104 circRNAs were significantly upregulated and 185 circRNAs downregulated in DR patients compared to DM patients (Figure 1C). In addition, 1,106 circRNAs were significantly upregulated and 1,346 circRNAs downregulated in DR patients, compared to healthy controls (Figure 1D).

### Functional Analysis of Differential circRNAs by Gene Ontology (GO) and Kyoto Encyclopedia of Genes and Genomes (KEGG) Analysis

**FIGURE 2:**
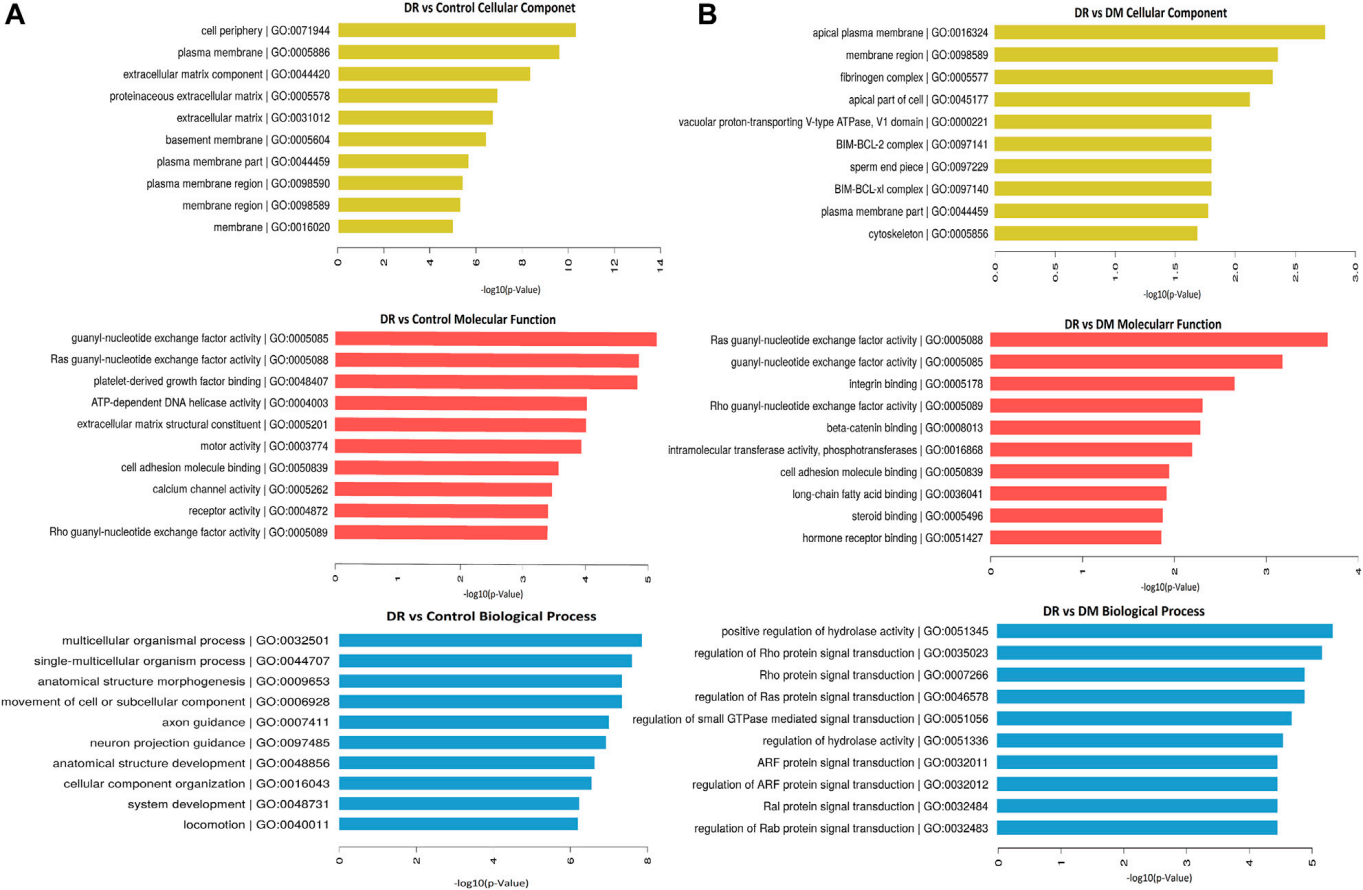
Differentially expressed circRNAs in DR samples by GO analysis. **(A)** DR vs control group, **(B)** DR vs DM group.

We analyzed the parent genes of differential circRNAs by using KEGG and GO analyses to predict their biological functions. The top 10 pathways enriched in DR patients were mainly related to cellular components, molecular functions, and biological processes. Those enriched signaling pathways were associated with cell periphery, plasma membrane, extracellular matrix component, proteinaceous extracellular matrix, and extracellular matrix (Figure 2A). In terms of molecular functions, guanyl nucleotide exchange factor activity and Ras guanyl nucleotide exchange factor activity were the most enriched aspects (Figure 2B). The most enriched biological functions included multicellular organismal process, single-multicellular organism process, anatomical structure morphogenesis, and the movement of cell or subcellular components, all of which were associated with cell growth and proliferation (Figure 2C).

**FIGURE 3:**
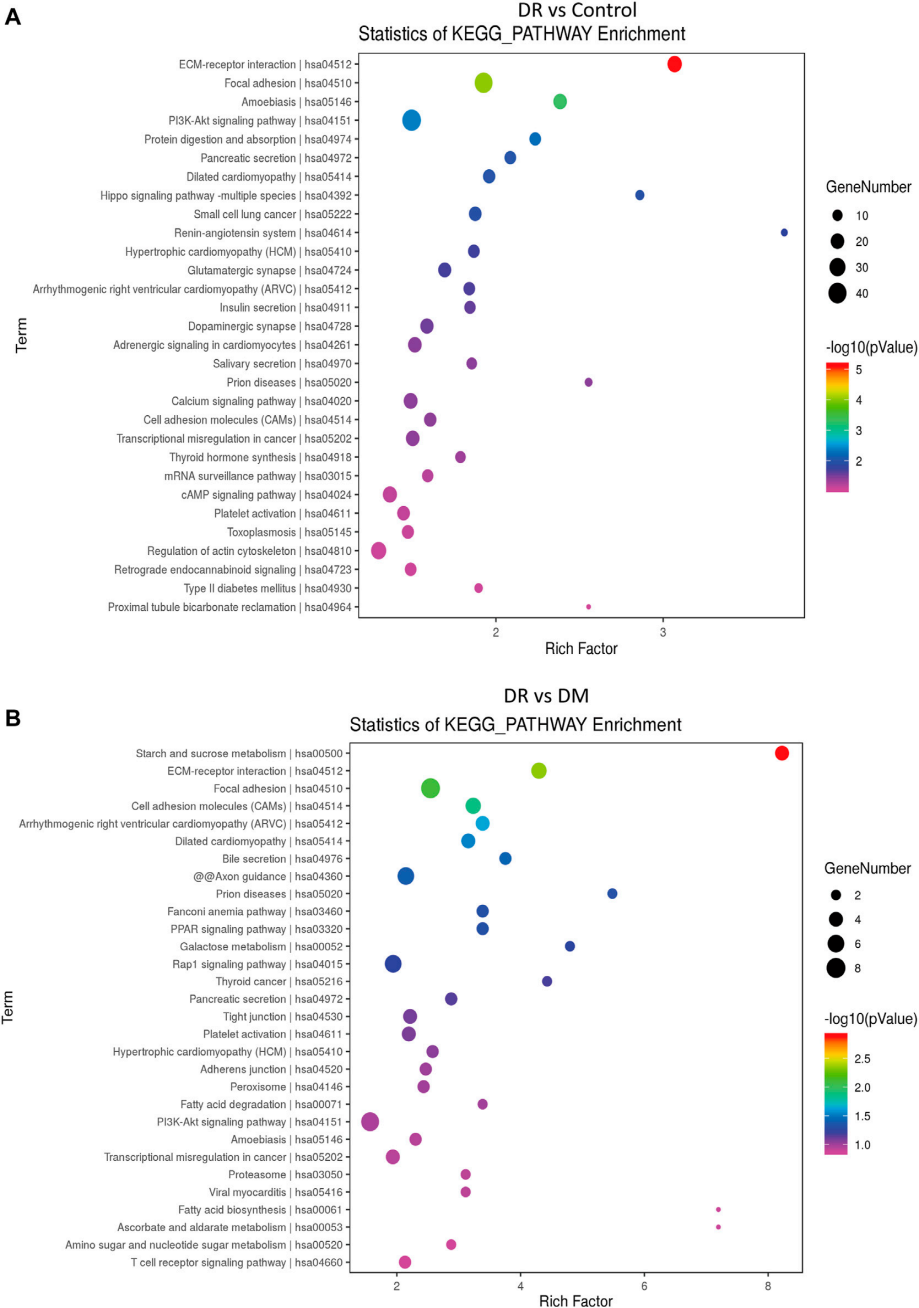
Differentially expressed circRNAs in DR patients by KEGG analysis. **(A)** DR vs control group, **(B)** DR vs. DM group.

In addition, KEGG pathway analysis showed the top 30 enriched pathways in DR patients compared to DM patients (Figure 3A) and to healthy controls (Figure 3B). Specifically, those differential circRNAs were mainly focused on ECM–receptor interaction and focal adhesion.

### Validation of circRNA Level by RT-qPCR

**FIGURE 4:**
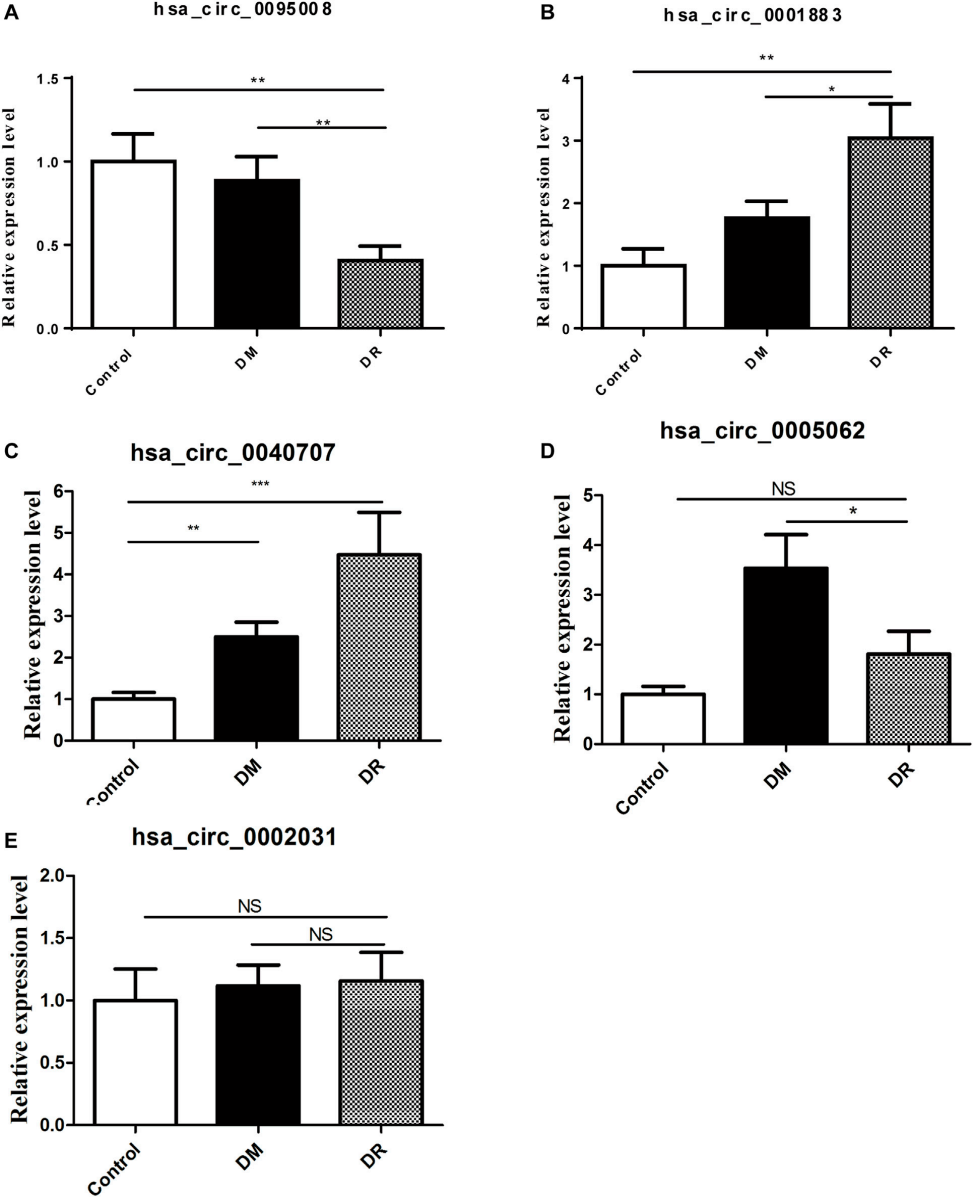
Validation of five candidate circRNAs in DR samples (*n* = 42) by RT-qPCR. **(A)** hsa_circ_0095008; **(B)** hsa_circ_0001883; **(C)** hsa_circ_0040707; **(D)** hsa_circ_0005062; **(E)** hsa_circ_0002031), compared with DM samples (*n* = 60) and controls (*n* = 20).*p < 0.05, **p < 0.01, ***p < 0.001.

To further identify biomarkers for DR patients, the top five upregulated or downregulated circRNAs (*p* ≤ 0.05 and raw processed signal ≥100) between DR and either DM or control groups are listed in Table 2. RT-qPCR technology was performed in an independent cohort (healthy controls, *n* = 20; DM patients without DR, *n* = 60; DR patients, *n* = 42) to detect these five candidate circRNAs (Figure 4). Detailed clinical characteristics of the study population are shown in Table 3. Trends of hsa_circ_0095008 and hsa_circ_0001883 were consistent with the results of circRNA arrays. hsa_circ_0095008 expression level was decreased by 2.18-fold (*p* = 0.013) and 2.47-fold (*p* = 0.001) in DR patients compared to DM patients and healthy controls, respectively (Figure 4A). hsa_circ_0001883 expression was increased by 1.73-fold (*p* = 0.025) and 3.04-fold (*p* = 0.015) in DR patients compared to DM and healthy controls, respectively (Figure 4B). A striking increase in hsa_circ_0040707 (*p* = 0.022) was identified in DR compared to healthy controls (Figure 4C). Moreover, the expression of hsa_circ_0005062 was significantly elevated (fold change = -1.953, *p* = 0.037) in DR patients, compared to DM patients (Figure 4D). However, there was no significant difference in hsa_circ_0002031 expression among these groups (Figure 4E).

**TABLE 2:** List of differentially expressed circRNAs in patients with DR.

circRNA ID	DR vs controls			DR vs DM		
	Fold change	p-value	Regulation	Fold change	p-value	Regulation
hsa_circ_0095008	3.302807	0.000866	Down	2.057623	0.040505	Down
hsa_circ_0005062	2.764961	0.009428	Down	4.017943	0.000528	Down
hsa_circ_0001883	3.217526	0.004683	Up	2.126805	0.041181	Up
hsa_circ_0040707	3.140882	0.019335	Up	3.287805	0.016502	Up
hsa_circ_0002031	2.379722	0.005157	Down	3.232438	0.042273	Down

**TABLE 3:** Detailed clinical parameters of study population.

	Controls	DM	DR
No.	20	60	42
Age (years)	41.35 ± 3.116	63.58 ± 1.344	65.511.271
Sex, female	9	35	22
BMI		24.35 ± 0.3988	23.72 ± 0.5683
HbA1c (%)		7.652 ± 0.1992	7.443 ± 0.2271
SBP (mmHg)		148.0 ± 2.570	144.2 ± 2.309
DBP (mmHg)		76.12 ± 1.965	74.29 ± 2.173
Glucose (mmol/L)		7.246 ± 0.2674	7.586 ± 0.3935
TCHO (mmol/L)		4.759 ± 0.1309	5.156 ± 0.1604
TG (mmol/L)		1.746 ± 0.1433	1.576 ± 0.1369
LDL (mmol/L)		1.255 ± 0.03701	1.275 ± 0.04632
HDL (mmol/L)		2.804 ± 0.1109	3.126 ± 0.1376
Tbil (umol/L)		12.90 ± 0.6937	12.94 ± 0.9465

BMI, body mass index; HbA1c, glycated hemoglobin A1c; SBP, systolic blood pressure; DBP, diastolic blood pressure; TCHO, total cholesterol; TG, triglyceride; LDL, low-density lipoprotein; LDL, low-density lipoprotein; TBil, total bilirubin.

### Diagnostic Value of Differentially Expressed circRNAs in PBMCs of DR Patients

**FIGURE 5:**
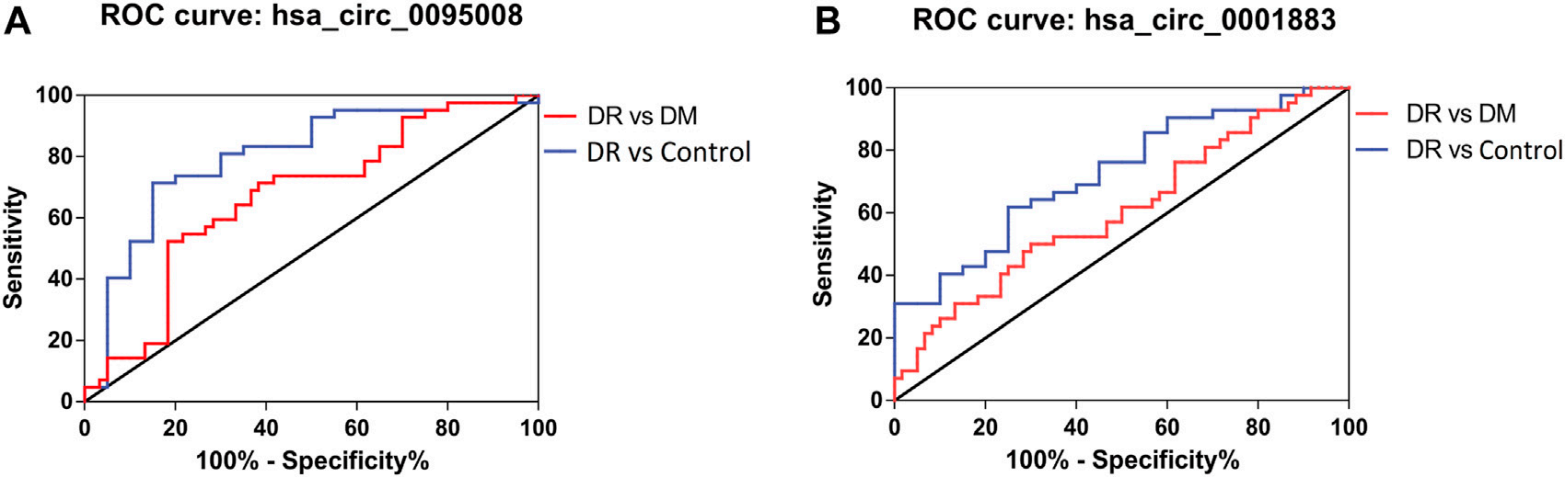
ROC analysis of hsa_circ_0095008 and hsa_circ_0001883 in DR samples. Diagnostic value of **(A)** hsa circ 0095008 and **(B)** hsa circ 0001883 for DR. Blue lines represent ROC curves of circRNAs for distinguishing DR patients from healthy controls ,while red lines represent ROC curves of circRNAs for distinguishing DR patients from DM patients without DR.

Receiver operating characteristic curve (ROC) was performed to analyze the sensitivity and specificity of circRNAs. The area under curve (AUC), cutoff value, sensitivity, and specificity of ROC analysis are listed in Table 4. These results showed that the AUCs of hsa_circ_0095008 were 0.671 (95% CI, 0.565–0.778) (*p* = 0.003) between DR and DM patients, and 0.804 (95% CI, 0.681–0.926) (*p* = 0.0001) between DR patients and healthy controls, respectively (Figure 5A). The AUCs of hsa_circ_0001883 were 0.607 (95% CI, 0.495–0.719) (*p* = 0.066) between DR and DM patients, and 0.725 (95% CI, 0.595–0.855) (*p* = 0.004) between DR patients and healthy controls (Figure 5B).

**TABLE 4:** AUC characteristics of hsa_circ_0095008 and hsa_circ_0001883.

Group	CircRNA ID	AUC	Sensitivity	Specificity	PPV	NPV
		(95% CI)	% (95%CI)	% (95%CI)	% (95%CI)	% (95%CI)
DR vs DM	hsa_circ_0095008	0.671	52.38	81.67	66.7	71
		0.565–0.778	36.42–68.00	69.56–90.48	50.6–82.8	60.3–81.7
	hsa_circ_0001883	0.607	50.00	70.00	52.5	66.1
		0.495–0.719	34.2–65.8	56.8–81.2	37–68	54.3–77.9
DR vs controls	hsa_circ_0095008	0.804	71.43	85	90.9	58.6
		0.681–0.926	55.42–84.28	62.11–96.79	81.1–100.7	40.7–76.5
	hsa_circ_0001883	0.725	61.90	75	81.25	46.7
		0.595–0.855	45.6–76.4	50.9–91.3	67.7–94.8	28.8–64.5

AUC, area under curve; PPV, positive predictive value; NPV, negative predictive value.

We further considered whether these circRNAs could serve as diagnostic biomarkers to distinguish DR from DM. The optimal cutoff value of hsa_circ_0095008 was 0.294, with a sensitivity of 52.38%, a specificity of 81.67%, a positive predictive value (PPV) of 66.7%, and a negative predictive value (NPV) of 71%. The cutoff value of hsa_circ_0001883 was 0.066, with a sensitivity of 50%, a specificity of 70%, a PPV of 52.5%, and an NPV of 66.1% for DR patients.

To diagnose DR from healthy controls, the sensitivity and specificity of hsa_circ_0095008 were 71.43 and 85%, respectively (cutoff value = 0.447, PPV = 90.9%, NPV = 58.6%); the sensitivity and specificity of hsa_circ_0001883 were 61.9 and 75%, respectively (cutoff value = 1.744, PPV = 81.25%, NPV = 46.7%). Taken together, hsa_circ_0095008 and hsa_circ_0001883 have diagnostic potential for DR.

### Construction of circRNA–miRNA Network

**FIGURE 6:**
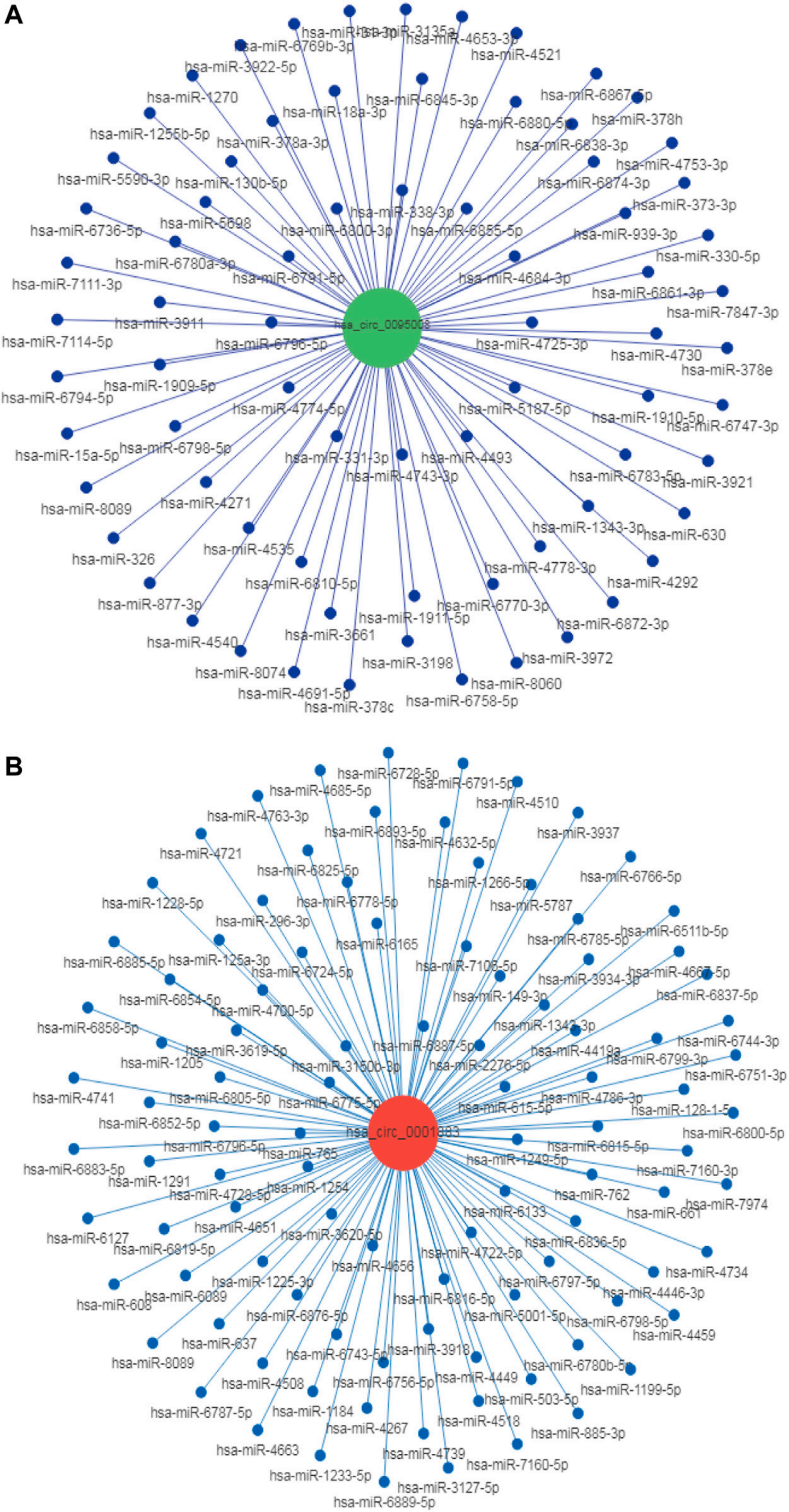
Target miRNA prediction of hsa_circ_0095008 and hsa_circ_0001883 in DR samples. The CircRNA/miRNA regulatory networks of **(A)** hsa_circ_0095008 and **(B)** hsa_circ_0001883 were predicted by miRanda- 3.3 software.The red and blue dots represent circRNAs and miRNAs, respectively.

It is well established that circRNAs have potential to regulate mRNA levels as miRNA sponges (Lyu and Huang, 2017). CircRNAs contain many miRNA binding sites and act as miRNA sponges to regulate the expression of miRNA targets (Ebbesen et al., 2017). For example, circRNA ciRS-7 contains 70 conserved binding sites for miR7. Thus, miR-7 levels can be increased by inhibiting the expression of circRNA ciRS-7 (Hansen et al., 2013). To explore whether hsa_circ_0095008 and hsa_circ_0001883 were miRNA sponges, we predicted their binding miRNAs using miRanda-3.3 software combined with entropy values below 20. Bioinformatics software Cytoscape was utilized to construct circRNA–miRNA networks. The prediction results demonstrated that they had more than 100 binding miRNAs, respectively, suggesting that hsa_circ_0095008 and hsa_circ_0001883 might regulate miRNA levels by acting as miRNA sponges in PBMCs (Figure 6). Among them, only 2 miRNAs had more than one binding site for hsa_circ_0095008, whereas there were 100 miRNAs with more than one binding site for hsa_circ_0001883. The current study also demonstrated that beyond acting as miRNA sponges, circRNAs could also serve as protein sponges or encode proteins to perform regulatory functions (Yang et al., 2018; Zhang et al., 2018; Huang et al., 2020; Wu et al., 2021). Thus, the additional roles of hsa_circ_0095008 and hsa_circ_0001883 in DR progression are still urgently needed to be further investigated.

## Discussion

As one of the most common diabetic complications, DR is more difficult to diagnose than others, considering that its early symptoms are not obvious and the clinical presentation is not very specific (Stitt et al., 2016). Generally, DR patients could be diagnosed only when irreversible eye damage occurred, including blurred vision and eventual blindness (Wong et al., 2016; Sheen et al., 2020). The increasing incidence of retinal neovascularization is the core factor contributing to DR (Li et al., 2018). However, the current therapeutic option for DR is still limited due to severe side effects (Sun and Jampol, 2019; Crabtree and Chang, 2021; Everett and Paulus, 2021). Therefore, it is crucial to improve the early diagnosis of DR.

CircRNAs, a class of circular non-coding RNAs, play vital biological functions in many physiological processes. CircRNAs were extensively explored as diagnostic and predictive biomarkers for various diseases, including cancer, neurological disease, inflammatory bowel disease, skin diseases, and Alzheimer’s disease (Ye et al., 2019; Zhang et al., 2019; Wang et al., 2020; Wu et al., 2020; Shengnan Li et al., 2021). To explore more sensitive biomarkers for DR screening, many samples extracted from different optical tissues, including retinal, vitreous humor, and serum, were studied (Gu et al., 2017; Jiang et al., 2020; He et al., 2020). He et al. studied the profile of circRNA expression in the vitreous humor of patients with proliferative diabetic retinopathy (PDR) (He et al., 2020). Gu et al. testified about many dysregulated circRNAs in serum from DR patients. hsa_circ_063981, hsa_circ_404457, hsa_circ_100750, hsa_circ_406918, hsa_circRNA_104387, hsa_circ_103410, and hsa_circ_100192 were significantly upregulated in DR patients compared to DM patients and healthy controls (Gu et al., 2017). Li et al. identified the profile of exosomal circRNAs in serum of PDR patients to explore their possible pro-angiogenic role (Xinsheng Li et al., 2021).

As it is difficult to obtain RNA from vitreous fluidand and serum exosome, and exon circRNA in serum are unstable, PBMCs are the ideal material for clinical diagnosis of DR (Wen et al., 2020). Hence, our study first identified the profile of differential circRNA in the PBMCs of healthy controls, DM patients, and DR patients. According to our outcomes represented in Figure 3, the differential circRNAs in PBMCs of DR patients had an important role in cell migration based on enrichment in ECM–receptor interaction and focal adhesion pathways. It was also confirmed by RT-qPCR in Figures 4A,B that hsa_circ_0095008 significantly decreased and hsa_circ_0001883 significantly upregulated in the DR compared to DM and control groups, which is consistent with the results of microarray analysis. It was summarized that these two abnormal circRNAs were closely associated with the occurrence of DR.

Interestingly, orosomucoid-1 (ORM1), the host gene of hsa_circ_0001883, is an acute phase response (APR) protein that regulates angiogenesis by stimulating VEGF (Luo et al., 2015). Increasing evidence suggests that retinal neuronal degeneration started in the early stages of DR (Srinivasan et al., 2017; Rolev et al., 2021), while hsa_circ_0095008 is spliced from neural cell adhesion molecule 2 (NCAM2) that modulates neuronal morphogenesis and differentiation (Parcerisas et al., 2021), which also indicates that circRNAs play a potential role in the early stages of DR. Moreover, circRNAs mainly exert their regulatory functions as miRNA sponges. They can affect mRNA stability, regulate host gene transcription, bind to RNA-binding proteins, and even directly translate proteins (Zhou and Kuang, 2021). It is reported that circSMARCA5 inhibits host gene transcription by interacting with the host gene (Xiaolong Xu et al., 2020).

However, our study has several limitations. First, more clinical samples are warranted to inspect the potential role of abnormal circRNAs as early diagnostic biomarkers of DR. Second, both the region and Asian species of samples also limit their representation and further diagnostic application. Last, the mechanism of these functional circRNAs in DR progression is still unclear.

In conclusion, our study provided a new sight for the pathological mechanism of DR and revealed the potential value of hsa_circ_0095008 and hsa_circ_0001883 as non-invasive biomarkers for the early diagnosis of DR.

## Data Availability

The original contributions presented in the study are publicly available. This data can be found here: NCBI, GSE193974, https://www.ncbi.nlm.nih.gov/geo/query/acc.cgi?acc=GSE193974.

## References

[B1] Altesha M. A. Ni T. Khan A. Liu K. Zheng X. (2019). Circular RNA in Cardiovascular Disease. J. Cel Physiol 234 (5), 5588–5600. 10.1002/jcp.27384 30341894

[B2] Antonetti D. A. Silva P. S. Stitt A. W. (2021). Current Understanding of the Molecular and Cellular Pathology of Diabetic Retinopathy. Nat. Rev. Endocrinol. 17 (4), 195–206. 10.1038/s41574-020-00451-4 33469209 PMC9053333

[B3] Cai H. Li Y. Niringiyumukiza J. D. Su P. Xiang W. (2019). Circular RNA Involvement in Aging: An Emerging Player with Great Potential. Mech. Ageing Dev. 178, 16–24. 10.1016/j.mad.2018.11.002 30513309

[B4] Cao M. Zhang L. Wang J.-H. Zeng H. Peng Y. Zou J. (2019). Identifying circRNA-Associated-ceRNA Networks in Retinal Neovascularization in Mice. Int. J. Med. Sci. 16 (10), 1356–1365. 10.7150/ijms.35149 31692917 PMC6818188

[B5] Chen X.-J. Li M.-L. Wang Y.-H. Mou H. Wu Z. Bao S. (2020). Abundant Neural circRNA Cdr1as Is Not Indispensable for Retina Maintenance. Front. Cel Dev. Biol. 8, 565543. 10.3389/fcell.2020.565543 PMC7677238 33240875

[B6] Chen X.-J. Zhang Z.-C. Wang X.-Y. Zhao H.-Q. Li M.-L. Ma Y. (2020). The Circular RNome of Developmental Retina in Mice. Mol. Ther. - Nucleic Acids 19, 339–349. 10.1016/j.omtn.2019.11.016 31877410 PMC6938940

[B7] Crabtree G. S. Chang J. S. (2021). Management of Complications and Vision Loss from Proliferative Diabetic Retinopathy. Curr. Diab Rep. 21 (9), 33. 10.1007/s11892-021-01396-2 34477996

[B8] Ebbesen K. K. Hansen T. B. Kjems J. (2017). Insights into Circular RNA Biology. RNA Biol. 14 (8), 1035–1045. 10.1080/15476286.2016.1271524 27982727 PMC5680708

[B9] Everett L. A. Paulus Y. M. (2021). Laser Therapy in the Treatment of Diabetic Retinopathy and Diabetic Macular Edema. Curr. Diab Rep. 21 (9), 35. 10.1007/s11892-021-01403-6 34487257 PMC8420141

[B10] Fang N. Ding G.-W. Ding H. Li J. Liu C. Lv L. (2021). Research Progress of Circular RNA in Gastrointestinal Tumors. Front. Oncol. 11, 665246. 10.3389/fonc.2021.665246 33937077 PMC8082141

[B11] Funatsu H. Noma H. Mimura T. Eguchi S. Hori S. (2009). Association of Vitreous Inflammatory Factors with Diabetic Macular Edema. Ophthalmology 116 (1), 73–79. 10.1016/j.ophtha.2008.09.037 19118698

[B12] Gang Chen G. Qian H.-M. Chen J. Wang J. Guan J.-T. Chi Z.-L. (2021). Whole Transcriptome Sequencing Identifies Key circRNAs, lncRNAs, and miRNAs Regulating Neurogenesis in Developing Mouse Retina. BMC genomics 22 (1), 779. 10.1186/s12864-021-08078-z 34717547 PMC8557489

[B13] George A. K. Master K. Majumder A. Homme R. P. Laha A. Sandhu H. S. (2019). Circular RNAs Constitute an Inherent Gene Regulatory axis in the Mammalian Eye and Brain. Can. J. Physiol. Pharmacol. 97 (6), 463–472. 10.1139/cjpp-2018-0505 30444648

[B14] Gonzalez-Casanova J. Schmachtenberg O. Martinez A. D. Sanchez H. A. Harcha P. A. Rojas-Gomez D. (2021). An Update on Connexin Gap Junction and Hemichannels in Diabetic Retinopathy. Int. J. Mol. Sci. 22 (6), 3194. 10.3390/ijms22063194 33801118 PMC8004116

[B15] Gu Y. Ke G. Wang L. Zhou E. Zhu K. Wei Y. (2017). Altered Expression Profile of Circular RNAs in the Serum of Patients with Diabetic Retinopathy Revealed by Microarray. Ophthalmic Res. 58 (3), 176–184. 10.1159/000479156 28817829

[B16] Gu S. Wang X. Shi L. Sun Q. Hu X. Gu Y. (2020). Health-related Quality of Life of Type 2 Diabetes Patients Hospitalized for a Diabetes-Related Complication. Qual. Life Res. 29 (10), 2695–2704. 10.1007/s11136-020-02524-3 32410144

[B17] Han J. Gao L. Dong J. Bai J. Zhang M. Zheng J. (2017). The Expression Profile of Developmental Stage-dependent Circular RNA in the Immature Rat Retina. Mol. Vis. 23, 457–469. 28761319 PMC5524268

[B18] Hansen T. B. Jensen T. I. Clausen B. H. Bramsen J. B. Finsen B. Damgaard C. K. (2013). Natural RNA Circles Function as Efficient microRNA Sponges. Nature 495 (7441), 384–388. 10.1038/nature11993 23446346

[B19] He M. Wang W. Yu H. Wang D. Cao D. Zeng Y. (2020). Comparison of Expression Profiling of Circular RNAs in Vitreous Humour between Diabetic Retinopathy and Non-diabetes Mellitus Patients. Acta Diabetol. 57 (4), 479–489. 10.1007/s00592-019-01448-w 31749049

[B20] Hsiao K.-Y. Sun H. S. Tsai S.-J. (2017). Circular RNA - New Member of Noncoding RNA with Novel Functions. Exp. Biol. Med. (Maywood) 242 (11), 1136–1141. 10.1177/1535370217708978 28485684 PMC5478007

[B21] Huang A. Zheng H. Wu Z. Chen M. Huang Y. (2020). Circular RNA-Protein Interactions: Functions, Mechanisms, and Identification. Theranostics 10 (8), 3503–3517. 10.7150/thno.42174 32206104 PMC7069073

[B22] Jiang Q. Liu C. Li C.-P. Xu S.-S. Yao M.-D. Ge H.-M. (2020). Circular RNA-Znf532 Regulates Diabetes-Induced Retinal Pericyte Degeneration and Vascular Dysfunction. J. Clin. Invest. 130 (7), 3833–3847. 10.1172/jci123353 32343678 PMC7324174

[B23] Kaining Chen K. Chen C. Li H. Yang J. Xiang M. Wang H. (2021). Widespread Translational Control Regulates Retinal Development in Mouse. Nucleic Acids Res. 49 (17), 9648–9664. 10.1093/nar/gkab749 34469513 PMC8464051

[B24] Kautzky-Willer A. Harreiter J. Pacini G. (2016). Sex and Gender Differences in Risk, Pathophysiology and Complications of Type 2 Diabetes Mellitus. Endocr. Rev. 37 (3), 278–316. 10.1210/er.2015-1137 27159875 PMC4890267

[B25] Li P. Chen D. Cui Y. Zhang W. Weng J. Yu L. (2018). Src Plays an Important Role in AGE-Induced Endothelial Cell Proliferation, Migration, and Tubulogenesis. Front. Physiol. 9, 765. 10.3389/fphys.2018.00765 29977209 PMC6021521

[B26] Lin Z. Long F. Zhao M. Zhang X. Yang M. (2020). The Role of Circular RNAs in Hematological Malignancies. Genomics 112 (6), 4000–4008. 10.1016/j.ygeno.2020.06.051 32634468

[B27] Liu C. Yao M.-D. Li C.-P. Shan K. Yang H. Wang J.-J. (2017). Silencing of Circular RNA-Znf609 Ameliorates Vascular Endothelial Dysfunction. Theranostics 7 (11), 2863–2877. 10.7150/thno.19353 28824721 PMC5562221

[B28] Luo Z. Lei H. Sun Y. Liu X. Su D.-F. (2015). Orosomucoid, an Acute Response Protein with Multiple Modulating Activities. J. Physiol. Biochem. 71 (2), 329–340. 10.1007/s13105-015-0389-9 25711902

[B29] Lyu D. Huang S. (2017). The Emerging Role and Clinical Implication of Human Exonic Circular RNA. RNA Biol. 14 (8), 1000–1006. 10.1080/15476286.2016.1227904 27588461 PMC5680672

[B30] Mellough C. B. Bauer R. Collin J. Dorgau B. Zerti D. Dolan D. W. P. (2019). An Integrated Transcriptional Analysis of the Developing Human Retina. Development 146 (2). 10.1242/dev.169474 PMC6361134 30696714

[B31] Mengxue Xu M. Xie F. Tang X. Wang T. Wang S. (2020). Insights into the Role of Circular RNA in Macrophage Activation and Fibrosis Disease. Pharmacol. Res. 156, 104777. 10.1016/j.phrs.2020.104777 32244027

[B32] Meng-Lan Li M.-L. Wang W. Jin Z.-B. (2021). Circular RNAs in the Central Nervous System. Front. Mol. Biosci. 8, 629593. 10.3389/fmolb.2021.629593 33816552 PMC8017125

[B33] Naqshbandi M. Harris S. B. Esler J. G. Antwi-Nsiah F. (2008). Global Complication Rates of Type 2 Diabetes in Indigenous Peoples: A Comprehensive Review. Diabetes Res. Clin. Pract. 82 (1), 1–17. 10.1016/j.diabres.2008.07.017 18768236

[B34] Parcerisas A. Ortega-Gascó A. Pujadas L. Soriano E. (2021). The Hidden Side of NCAM Family: NCAM2, a Key Cytoskeleton Organization Molecule Regulating Multiple Neural Functions. Int. J. Mol. Sci. 22 (18). 10.3390/ijms221810021 PMC8471948 34576185

[B35] Rolev K. D. Shu X.-s. Ying Y. (2021). Targeted Pharmacotherapy against Neurodegeneration and Neuroinflammation in Early Diabetic Retinopathy. Neuropharmacology 187, 108498. 10.1016/j.neuropharm.2021.108498 33582150

[B36] Shan K. Liu C. Liu B.-H. Chen X. Dong R. Liu X. (2017). Circular Noncoding RNA HIPK3 Mediates Retinal Vascular Dysfunction in Diabetes Mellitus. Circulation 136 (17), 1629–1642. 10.1161/circulationaha.117.029004 28860123

[B37] Sheen Y.-J. Kung P.-T. Sheu W. H.-H. Kuo W.-Y. Tsai W.-C. (2020). Impact of Liver Cirrhosis on Incidence of Dialysis Among Patients with Type 2 Diabetes. Diabetes Ther. 11 (11), 2611–2628. 10.1007/s13300-020-00919-6 32901421 PMC7547941

[B38] Shengnan Li S. Hu W. Deng F. Chen S. Zhu P. Wang M. (2021). Identification of Circular RNA Hsa_circ_0001599 as a Novel Biomarker for Large-Artery Atherosclerotic Stroke. DNA Cel Biol. 40 (3), 457–468. 10.1089/dna.2020.5662 33493415

[B39] Srinivasan S. Dehghani C. Pritchard N. Edwards K. Russell A. W. Malik R. A. (2017). Corneal and Retinal Neuronal Degeneration in Early Stages of Diabetic Retinopathy. Invest. Ophthalmol. Vis. Sci. 58 (14), 6365–6373. 10.1167/iovs.17-22736 29260193

[B40] Stitt A. W. Curtis T. M. Chen M. Medina R. J. McKay G. J. Jenkins A. (2016). The Progress in Understanding and Treatment of Diabetic Retinopathy. Prog. Retin. Eye Res. 51, 156–186. 10.1016/j.preteyeres.2015.08.001 26297071

[B41] Sun J. K. Jampol L. M. (2019). The Diabetic Retinopathy Clinical Research Network (DRCR.Net) and its Contributions to the Treatment of Diabetic Retinopathy. Ophthalmic Res. 62 (4), 225–230. 10.1159/000502779 31554001

[B42] Sun L.-F. Zhang B. Chen X.-J. Wang X.-Y. Zhang B.-W. Ji Y.-Y. (2019). Circular RNAs in Human and Vertebrate Neural Retinas. RNA Biol. 16, 821–829. 10.1080/15476286.2019.1591034 30874468 PMC6546369

[B43] Sun L. F. Chen X. J. Jin Z. B. (2020). Emerging Roles of Non-coding RNAs in Retinal Diseases: A Review. Clin. Exp. Ophthalmol 48 (8), 1085–1101. 10.1111/ceo.13806 32519377

[B44] Sun L. F. Ma Y. Ji Y. Y. Wu Z. Wang Y. H. Mou H. (2021). Circular Rims2 Deficiency Causes Retinal Degeneration. Adv. Biol. (Weinh) 5 (12), e2100906. 10.1002/adbi.202100906 34738746

[B45] Wang Y. Mo Y. Gong Z. Yang X. Yang M. Zhang S. (2017). Circular RNAs in Human Cancer. Mol. Cancer 16 (1), 25. 10.1186/s12943-017-0598-7 28143578 PMC5282898

[B46] Wang J.-J. Shan K. Liu B.-H. Liu C. Zhou R.-M. Li X.-M. (2018). Targeting Circular RNA-ZRANB1 for Therapeutic Intervention in Retinal Neurodegeneration. Cell Death Dis 9 (5), 540. 10.1038/s41419-018-0597-7 29748605 PMC5945597

[B47] Wang M. Gu B. Yao G. Li P. Wang K. (2020). Circular RNA Expression Profiles and the Pro-tumorigenic Function of CircRNA_10156 in Hepatitis B Virus-Related Liver Cancer. Int. J. Med. Sci. 17 (10), 1351–1365. 10.7150/ijms.45637 32624692 PMC7330659

[B48] Wen G. X. Zhou T. Gu W. (2020). The Potential of Using Blood Circular RNA as Liquid Biopsy Biomarker for Human Diseases. Protein Cell 12, 911–946. 10.1007/s13238-020-00799-3 33131025 PMC8674396

[B49] Wong T. Y. Cheung C. M. G. Larsen M. Sharma S. Simó R. (2016). Diabetic Retinopathy. Nat. Rev. Dis. Primers 2, 16012. 10.1038/nrdp.2016.12 27159554

[B50] Wu X. Xiao Y. Ma J. Wang A. (2020). Circular RNA: A Novel Potential Biomarker for Skin Diseases. Pharmacol. Res. 158, 104841. 10.1016/j.phrs.2020.104841 32404296

[B51] Wu N. Xu J. Du W. W. Li X. Awan F. M. Li F. (2021). YAP Circular RNA, circYap, Attenuates Cardiac Fibrosis via Binding with Tropomyosin-4 and Gamma-Actin Decreasing Actin Polymerization. Mol. Ther. 29 (3), 1138–1150. 10.1016/j.ymthe.2020.12.004 33279723 PMC7934790

[B52] Xiaolong Xu X. Zhang J. Tian Y. Gao Y. Dong X. Chen W. (2020). CircRNA Inhibits DNA Damage Repair by Interacting with Host Gene. Mol. Cancer 19, 128. 10.1186/s12943-020-01246-x 32838810 PMC7446195

[B53] Xinsheng Li X. Wang J. Qian H. Wu Y. Zhang Z. Hu Z. (2021). Serum Exosomal Circular RNA Expression Profile and Regulative Role in Proliferative Diabetic Retinopathy. Front. Genet. 12, 719312. 10.3389/fgene.2021.719312 34447414 PMC8383346

[B54] Yang Y. Gao X. Zhang M. Yan S. Sun C. Xiao F. (2018). Novel Role of FBXW7 Circular RNA in Repressing Glioma Tumorigenesis. J. Natl. Cancer Inst. 110 (3), 304–315. 10.1093/jnci/djx166 PMC6019044 28903484

[B55] Ye Y.-L. Yin J. Hu T. Zhang L.-P. Wu L.-Y. Pang Z. (2019). Increased Circulating Circular RNA_103516 Is a Novel Biomarker for Inflammatory Bowel Disease in Adult Patients. World J. Gastroenterol. 25 (41), 6273–6288. 10.3748/wjg.v25.i41.6273 31749597 PMC6848015

[B56] Zhang Y. Zhang X.-O. Chen T. Xiang J.-F. Yin Q.-F. Xing Y.-H. (2013). Circular Intronic Long Noncoding RNAs. Mol. Cel 51 (6), 792–806. 10.1016/j.molcel.2013.08.017 24035497

[B57] Zhang S.-J. Chen X. Li C.-P. Li X.-M. Liu C. Liu B.-H. (2017). Identification and Characterization of Circular RNAs as a New Class of Putative Biomarkers in Diabetes Retinopathy. Invest. Ophthalmol. Vis. Sci. 58 (14), 6500–6509. 10.1167/iovs.17-22698 29288268

[B58] Zhang M. Huang N. Yang X. Luo J. Yan S. Xiao F. (2018). A Novel Protein Encoded by the Circular Form of the SHPRH Gene Suppresses Glioma Tumorigenesis. Oncogene 37 (13), 1805–1814. 10.1038/s41388-017-0019-9 29343848

[B59] Zhang Y. Yu F. Bao S. Sun J. (2019). Systematic Characterization of Circular RNA-Associated CeRNA Network Identified Novel circRNA Biomarkers in Alzheimer's Disease. Front. Bioeng. Biotechnol. 7, 222. 10.3389/fbioe.2019.00222 31572720 PMC6749152

[B60] Zheng Y. Ley S. H. Hu F. B. (2018). Global Aetiology and Epidemiology of Type 2 Diabetes Mellitus and its Complications. Nat. Rev. Endocrinol. 14 (2), 88–98. 10.1038/nrendo.2017.151 29219149

[B61] Zhou H.-r. Kuang H.-y. (2021). Circular RNAs: Novel Target of Diabetic Retinopathy. Rev. Endocr. Metab. Disord. 22, 205–216. 10.1007/s11154-021-09646-0 33761053

[B62] Zhu Z. Duan P. Song H. Zhou R. Chen T. (2021). Downregulation of Circular RNA PSEN1 Ameliorates Ferroptosis of the High Glucose Treated Retinal Pigment Epithelial Cells via miR-200b-3p/cofilin-2 axis. Bioengineered 12 (2), 12555–12567. 10.1080/21655979.2021.2010369 34903141 PMC8809929

